# Accuracy of the Mini-Mental State Examination and Montreal Cognitive Assessment in Detecting Cognitive Impairment in Older Adults: A Comparative Study Adjusted for Educational Level

**DOI:** 10.3390/neurosci6030086

**Published:** 2025-09-05

**Authors:** Paula Andreatta Maduro, Leandro Paim da Cruz Carvalho, Luiz Alcides Ramires Maduro, Ana Beatriz da Costa Rodrigues, Alaine Souza Lima Rocha, Lilian Ramine Ramos de Souza Matos, Marcelo de Maio Nascimento, Bruno Bavaresco Gambassi, Paulo Adriano Schwingel

**Affiliations:** 1 Programa de Pós-Graduação em Ciências da Saúde (PPGCS), Universidade de Pernambuco (UPE), Recife 50100-130, PE, Brazil; paula.maduro@ebserh.gov.br (P.A.M.); alaine.rocha@ufc.br (A.S.L.R.); 2 Laboratório de Pesquisas em Desempenho Humano (LAPEDH), Universidade de Pernambuco (UPE), Petrolina 56328-900, PE, Brazil; leandro.paim@upe.br (L.P.d.C.C.); luiz.maduro@univasf.edu.br (L.A.R.M.); anabeatriz98rodrigues@gmail.com (A.B.d.C.R.); professorbrunobavaresco@gmail.com (B.B.G.); 3 Hospital de Ensino Washington Antônio de Barros da Universidade Federal do Vale do São Francisco (HU-UNIVASF), Empresa Brasileira de Serviços Hospitalares (EBSERH), Petrolina 56304-205, PE, Brazil; lilian.ramos@ebserh.gov.br; 4 Programa de Pós-Graduação em Reabilitação e Desempenho Funcional (PPGRDF), Universidade de Pernambuco (UPE), Petrolina 56328-900, PE, Brazil; 5 Colegiado de Educação Física (CEFIS), Universidade Federal do Vale do São Francisco (UNIVASF), Petrolina 56304-917, PE, Brazil; marcelo.nascimento@univasf.edu.br; 6 Departamento de Fisioterapia, Universidade Federal do Ceará (UFC), Fortaleza 60440-900, CE, Brazil; 7 Programa de Pós-Graduação em Gestão e Atenção à Saúde (PPGGAS), Universidade Ceuma (UNICEUMA), São Luís 65075-120, MA, Brazil

**Keywords:** cognitive screening, mild cognitive impairment, educational adjustment, ROC curve analysis, mini-mental state examination, Montreal cognitive assessment

## Abstract

Early detection of cognitive decline in older adults is essential for implementing timely interventions. This study aimed to compare the diagnostic accuracy of the Mini-Mental State Examination (MMSE^®^) and the Montreal Cognitive Assessment (MoCA©) in identifying cognitive impairment among community-dwelling older adults, while considering the effect of educational level. A cross-sectional, analytical study was conducted with 90 individuals aged 60 years or older, classified into cognitively preserved and cognitively impaired groups using the Clinical Dementia Rating (CDR) scale. Cognitive performance was assessed using the MMSE and MoCA, with results analyzed using both standard and education-adjusted cut-off scores. Diagnostic accuracy was evaluated using Receiver Operating Characteristic (ROC) curves. The MoCA demonstrated superior discriminative ability compared to the MMSE, with a significantly larger area under the ROC curve (AUC = 0.943 vs. 0.826; *p* < 0.001), higher sensitivity (90.2% vs. 78.4%), and higher specificity (87.2% vs. 76.9%). When education-adjusted cut-off scores were applied, the MoCA achieved markedly improved diagnostic accuracy (87.8%) compared to the MMSE (71.1%), with stronger agreement with CDR classifications (κ = 0.746 vs. κ = −0.132). These findings demonstrate that the MoCA is more sensitive in detecting cognitive impairment and should be considered the preferred screening tool in clinical and research settings, particularly when appropriate educational adjustments are applied.

## 1. Introduction

Dementia is one of the leading causes of disability and dependency among older adults worldwide, and its prevalence continues to rise due to global population aging [1,2]. An estimated 55 million people currently live with dementia, with over 60% residing in low- and middle-income countries [1]. This growing burden underscores the urgent need for early detection strategies, as timely identification of cognitive impairment can facilitate interventions that preserve function and improve quality of life [3].

Neuropsychological screening tools play a pivotal role in the early identification of cognitive decline [4]. Among these, the Mini-Mental State Examination—MMSE^®^ (Psychological Assessment Resources [PAR], Inc., Lutz, FL, United States of America [USA]) [5] is the most widely used and validated instrument in clinical and research settings [6,7]. Its ease of administration and broad application contribute to its utility in routine screening. However, several studies have raised concerns about its sensitivity in detecting early-stage dementia, particularly among individuals with low educational attainment [8,9,10].

To address these limitations, alternative tools such as the Montreal Cognitive Assessment—MoCA© (MoCA Test Inc., Greenfield Park, QC, Canada) [11] have been developed. The MoCA offers broader coverage of cognitive domains, including executive function, visuospatial ability, and memory, and has demonstrated superior sensitivity in detecting mild cognitive impairment [12,13,14]. Comparative studies indicate that the MoCA may outperform the MMSE in various populations, showing stronger correlations with comprehensive neuropsychological batteries [15,16,17].

Despite this evidence, no consensus exists regarding optimal cut-off scores for these instruments, and performance may vary significantly based on sociodemographic factors such as educational level [11,13]. Moreover, few studies have simultaneously examined the diagnostic accuracy of both the MMSE and MoCA using educational adjustments in diverse older adult populations, highlighting the need for further comparative validation [14,18].

Therefore, this preliminary study aims to evaluate and compare the diagnostic accuracy of the MMSE and MoCA in identifying cognitive impairment in community-dwelling older adults. Importantly, the analysis includes comparisons of standard and education-adjusted cut-off scores to determine which approach provides greater sensitivity and specificity for early detection. These findings may guide future clinical decision-making and contribute to improving cognitive screening strategies in primary care and geriatric settings.

## 2. Materials and Methods

### 2.1. Study Design and Participants

This comparative analytical study was conducted between January and September 2022 with a sample of 90 community-dwelling older adults (aged 60 years or older) receiving care at the University Hospital of the Federal University of the São Francisco Valley (HU-UNIVASF), Petrolina, PE, Brazil, managed by the Brazilian Company of Hospital Services (EBSERH), Brasília, DF, Brazil. The participants for this study were selected from a larger cohort of 101 older adults, whose recruitment and primary characteristics have been described previously.

For the present analysis, a final sample of 90 participants who had complete data for both the MMSE and MoCA assessments was included [3]. The study followed the translated and updated versions of the Strengthening the Reporting of Observational Studies in Epidemiology (STROBE) statement [19,20], adhering to international guidelines for reporting observational research [21].

The required sample size was estimated using G*Power software (Heinrich-Heine-Universität Düsseldorf, Düsseldorf, Germany, version 3.1.9.4, 2019). For a two-tailed independent samples *t*-test with an expected medium effect size (Cohen’s *d* = 0.50), α = 0.05, and statistical power (1–β) of 0.80, a minimum of 64 participants (32 per group) was required.

To account for potential data loss or ineligibility, the sample was increased by approximately 40%, resulting in a final sample of 90 older adults. This sample size was sufficient to detect statistically meaningful differences in cognitive performance and diagnostic accuracy between groups. Furthermore, this sample size, comprising 51 cases and 39 controls, is also considered sufficient to yield stable and reliable estimates in the Receiver Operating Characteristic (ROC) curve analysis for a test with high expected accuracy [22].

Eligible participants included male and female outpatients aged ≥60 years, with at least four years of formal education, regardless of marital status or family income. This educational criterion was established to ensure a baseline level of literacy, thereby minimizing the risk of floor effects and ensuring that the assessments reflected cognitive function rather than literacy skills, a known confounder in populations with heterogeneous educational backgrounds. Recruitment occurred at the HU-UNIVASF Polyclinic outpatient center.

Exclusion criteria were established to minimize potential confounding factors. We excluded individuals with: (a) a diagnosis of Parkinson’s disease; (b) a history of stroke or transient ischemic attack; (c) uncorrected motor or sensory deficits that could interfere with neuropsychological assessment; (d) a history of significant cardiovascular events (e.g., acute myocardial infarction, angina, invasive cardiovascular procedures); (e) use of psychotropic drugs or beta-blockers; (f) use of four or more antihypertensive medications; (g) systolic blood pressure (SBP) ≥ 180 mmHg or diastolic blood pressure (DBP) ≥ 110 mmHg; and (h) untreated hypothyroidism. Furthermore, to avoid the confounding effects of depressive symptoms on cognitive performance, individuals with a score > 18 on the Beck Depression Inventory (BDI), indicative of moderate-to-severe depression, were also excluded.

Cognitive status was determined by an experienced geriatric psychiatrist familiar with the local population, using the Clinical Dementia Rating (CDR) scale [23,24]. Participants with CDR = 0 were classified as cognitively preserved and allocated to the control group (n = 39), while those scoring between 0.5 and 2 were categorized as cognitively impaired (n = 51).

This research received ethical approval from the Research Ethics Committee of the School of Medical Sciences of Pernambuco (Report No. 4.389.686; CAAE: 38942320.4.0000.5192). All procedures were conducted in accordance with the ethical standards outlined in the Declaration of Helsinki (1964, revised in 2013) and complied with the Brazilian National Health Council Resolutions 466/2012 and 510/2016. Written informed consent was obtained from all individuals before they participated in the study.

### 2.2. Sociodemographic and Health Status Assessment

Sociodemographic and economic data were collected using standardized questionnaires based on Brazilian Institute of Geography and Statistics (IBGE) criteria. Variables included age, sex, marital status, race/ethnicity (self-reported), occupation, educational attainment, and household income (expressed in minimum wages).

General health status was assessed using a structured questionnaire encompassing medical history, current health conditions, and medication use. To ensure consistency and eliminate inter-rater variability, all cognitive assessments and interviews were administered by a single researcher (P.A.M.), a Ph.D. in Health Sciences with a research focus on geriatrics. The assessor holds official certification for the administration and scoring of the MoCA, as provided by MoCA Test Inc. For the MMSE, the assessor underwent a standardized training protocol under the direct supervision of the hospital’s lead clinical psychologist to ensure adherence to the established administration guidelines.

### 2.3. Functional and Clinical Evaluation

Functional assessment was conducted using the Katz Index of Independence in Activities of Daily Living [25] and the Lawton Instrumental Activities of Daily Living Scale [26], with the latter adapted for use in the Brazilian older adult population [27].

The Katz index evaluates an individual’s level of independence in performing basic activities of daily living (ADLs). It assesses six self-care tasks, arranged hierarchically by complexity: bathing, dressing, toileting, transferring, continence, and feeding. Each task is scored dichotomously—independent (1 point) or dependent (0 points)—yielding a total score ranging from 0 to 6 [25]. A score of 6 indicates full independence, whereas a score of 0 reflects total dependence across all assessed activities.

In contrast, the Lawton Scale evaluates an individual’s ability to perform instrumental activities of daily living (IADLs), which require greater cognitive and physical capacity [26]. The version used in this study was the Brazilian adaptation [27], comprising nine items that assess competencies such as telephone use, shopping, food preparation, housekeeping, laundry, transportation, medication management, financial handling, and minor household repairs. Scores range from 9 to 27, with higher scores indicating greater functional independence.

### 2.4. Cognitive Assessment

Cognitive function was assessed using the MMSE^®^ and the MoCA©. The MMSE evaluates multiple cognitive domains, including orientation, memory, attention, language, and visuoconstructional skills [5]. Cognitive impairment was initially classified based on the standardized cut-off score of <24 points [5]. Additionally, we employed education-adjusted cut-off scores recommended by Brucki et al. [28]: 25 points for individuals with 1–4 years of education, 26.5 points for those with 5–8 years, 28 points for those with 9–11 years, and 29 points for participants with ≥12 years of formal education.

The MoCA was used to provide a more comprehensive assessment of cognitive abilities, particularly executive function, abstraction, memory, attention, visuospatial skills, naming, orientation, and language [11]. The use of the MoCA in the Brazilian population, particularly those with varied educational backgrounds, is supported by studies that provide normative data for this demographic [29]. The standard cut-off score for cognitive impairment detection was <26 points, with one additional point added to scores for individuals with ≤12 years of education [11]. Furthermore, education-adjusted cut-off values proposed by Pinto et al. [30] were also applied: scores ≤21 points for participants with ≥12 years of schooling and ≤20 points for those with 4–12 years of education were considered indicative of cognitive impairment.

The present study utilized the official version of the MoCA©, with copyright permission granted by MoCA Test Inc. for its use. For the MMSE^®^, standardized forms were commercially procured from a Brazilian vendor officially authorized by the copyright holder, Psychological Assessment Resources, Inc. (PAR, Inc.), Florida, USA. This procedure ensured the legitimate use of both copyrighted psychological assessment tools.

### 2.5. Statistical Analysis

Data were double-entered into the Statistical Package for the Social Sciences (SPSS Inc., Chicago, IL, USA, release 16.0.2, 2007) to ensure accuracy and consistency. Descriptive statistics were calculated to characterize the sample: categorical variables were presented as absolute frequencies (*n*) and percentages (%), and continuous variables as means ± standard deviations (SD). Normality of distribution was verified using the Kolmogorov–Smirnov test. Between-group comparisons were conducted using independent samples *t*-tests for continuous variables. Pearson’s chi-square test (χ^2^) or Fisher’s exact test was employed as appropriate for categorical variables.

Diagnostic accuracy for MMSE and MoCA was evaluated through ROC curve analyses. The areas under the ROC curve (AUC) were calculated along with their respective 95% confidence intervals (95% CIs) and standard errors (SE). Sensitivity, specificity, positive predictive values (PPV), and negative predictive values (NPV) were computed for both screening instruments, considering standard and education-adjusted cut-off scores. Agreement between the cognitive impairment classification based on MMSE/MoCA scores and the CDR classification (reference standard) was assessed using Cohen’s kappa (κ). Interpretation of κ values was as follows: <0.40 indicated poor agreement, 0.41–0.60 moderate agreement, 0.61–0.80 good agreement, and >0.80 very good agreement. All *p*-values and 95% CIs were calculated and reported with exact values. A two-tailed significance level of 5% (*p* ≤ 0.05) was adopted for all statistical tests.

## 3. Results

The study included 90 older adults ranging from 60 to 90 years of age, with a mean age of 69.0 ± 6.5 years. Most participants were female (n = 62; 68.9%) and reported a monthly income equal to or lower than two minimum wages (n = 64; 71.1%). Participants with cognitive impairment were significantly older (*p* < 0.001), had fewer years of education (*p* = 0.001), and reported lower monthly income (*p* = 0.026) compared to those without cognitive impairment (Table 1). Additionally, the cognitively impaired group presented lower functional independence, demonstrated by poorer scores on both ADL and IADL assessments (both *p* < 0.001). MMSE and MoCA cognitive screening scores were also significantly lower among participants with cognitive impairment (*p* < 0.001).

**Figure 1:**
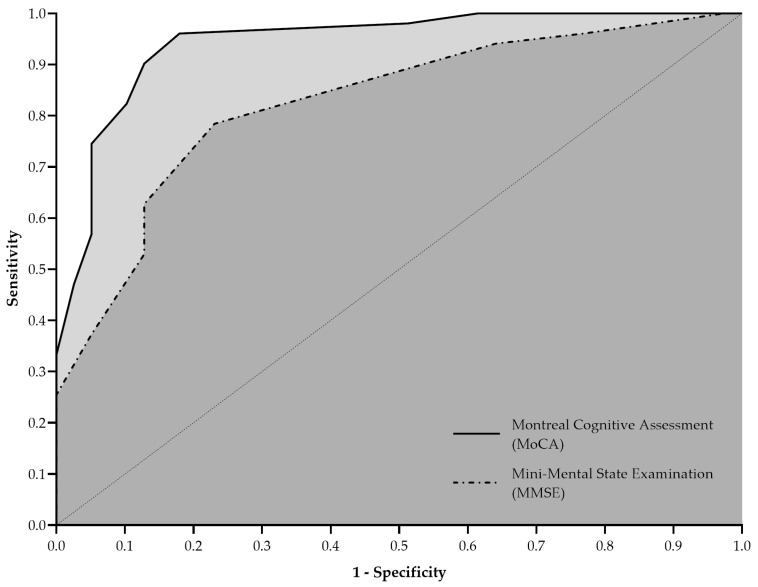
Receiver operating characteristic (ROC) curves comparing the accuracy of the mini-mental state examination (dashed line) and Montreal cognitive assessment (solid line) in identifying cognitive impairment in older adults.

Figure 1 illustrates the ROC curves for MMSE and MoCA. The MMSE yielded an AUC of 0.826 (95% CI: 0.740–0.911; SE: 0.044; *p* < 0.001), with sensitivity and specificity of 78.4% (95% CI: 65.4–87.5%) and 76.9% (95% CI: 61.7–87.4%), respectively. MoCA demonstrated superior discriminative ability, presenting an AUC of 0.943 (95% CI: 0.896–0.991; SE: 0.024; *p* < 0.001), with higher sensitivity (90.2%; 95% CI: 79.0–95.7%) and specificity (87.2%; 95% CI: 73.3–94.4%).

Using standardized cut-off scores, the overall diagnostic accuracy for cognitive impairment was 73.3% (95% CI: 63.0–82.1%) for MMSE and 62.2% (95% CI: 51.4–72.2%) for MoCA (Table 2). MMSE exhibited a PPV of 86.5% (95% CI: 73.3–93.7%) and an NPV of 64.1% (95% CI: 55.1–72.3%), whereas MoCA displayed a PPV of 60% (95% CI: 57.1–62.8%) and an NPV of 100% (95% CI: 47.8–100%). When cut-off scores were adjusted according to years of education, the MMSE classified 70% (n = 63) of older adults as cognitively impaired, compared to 64.4% (n = 58) classified by the MoCA. With education-adjusted cut-off scores, MoCA accuracy improved markedly to 87.8% (95% CI: 79.2–93.7%), surpassing MMSE accuracy of 71.1% (95% CI: 60.6–80.2%). The PPV and NPV values were notably higher for the MoCA (PPV = 84.5% [95% CI: 75.4–90.6%]; NPV = 93.8% [95% CI: 79.2–98.3%]) compared to the MMSE (PPV = 69.8 [95% CI: 62.2–74.5%]; NPV = 74.1% [95% CI: 57.4–85.8%]).

Finally, the agreement between cognitive screening tests and CDR varied considerably. The MMSE showed moderate agreement with standardized cut-off scores (κ = 0.479), but poor agreement when using education-adjusted cut-offs (κ = −0.132). In contrast, the MoCA demonstrated poor agreement with standardized cut-off scores (κ = −0.053) but good agreement (κ = 0.746) when adjusted for educational level, highlighting the importance of education-adjusted criteria.

## 4. Discussion

This study demonstrated that the MoCA outperformed the MMSE in screening cognitive impairment among older adults, exhibiting superior diagnostic accuracy in terms of both sensitivity (74.5% vs. 62.8%) and specificity (94.9% vs. 87.2%). Furthermore, the MoCA achieved an AUC above 0.90, indicating excellent discriminative capacity for detecting early neurocognitive decline. These findings align closely with previous evidence suggesting that the MoCA is more sensitive than MMSE in detecting subtle cognitive deficits, particularly in the initial stages of impairment [31,32,33,34].

A noteworthy finding from our analysis is the negative kappa value observed for both the MMSE with education-adjusted cut-off scores and the MoCA with its standard cut-off score. A negative kappa statistic indicates that the observed agreement between the screening test and the CDR was worse than the agreement expected by chance alone. This result does not invalidate the MMSE as a screening tool in general; rather, it highlights the critical failure of the specific adjustment strategy applied [28] within our particular sample. This underscores the significant risk of misclassification when applying normative data that may not be suitable for a given clinical population. Consequently, our findings reinforce the observation that the AUC from the ROC analysis provides a more robust and reliable measure of overall diagnostic accuracy, as it is independent of any single, potentially arbitrary, cut-off point.

Several factors are likely to contribute to the superior performance of the MoCA. This instrument includes a broader assessment of executive functions, attention, visuospatial abilities, and abstraction—domains often impaired early in cognitive decline [11]. Previous research has consistently highlighted that the MoCA contains tasks with a higher difficulty level, effectively capturing mild cognitive impairment (MCI) cases that the MMSE might overlook [35,36]. Moreover, Nagaratnam et al. [37] noted that the MMSE tends to have limited sensitivity and is best suited for assessing more advanced cognitive impairment or dementia. In contrast, the MoCA is advantageous for detecting mild and subtle changes across multiple cognitive domains.

The use of education-adjusted cut-off scores is a crucial methodological point that warrants emphasis, as it significantly increases screening accuracy in populations with diverse educational backgrounds [38,39]. Formal education is one of the primary indicators of cognitive reserve, a concept postulating that stimulating life experiences, such as education, can build greater brain resilience against the effects of neurodegenerative pathologies [30]. Ignoring the influence of education can lead to substantial classification bias: individuals with low educational attainment are at risk of being incorrectly classified as having cognitive impairment (false positives), while those with high academic attainment may have their initial decline masked by their cognitive reserve, resulting in normal screening outcomes [38,39]. Therefore, adjusting cut-off scores, as performed in this study, is not merely a statistical correction but a fundamental step to ensure the equity and ecological validity of screening instruments, making them fairer and more clinically useful in real-world contexts, such as that of Brazil. This is particularly relevant in the Brazilian context, where studies have shown that the MoCA’s accuracy can be limited in populations with very low educational levels, reinforcing the need for validated, education-adjusted norms to avoid misclassification [29].

To further contextualize our findings, we have integrated recent normative studies as suggested. Regarding the MoCA, the work of Pugh et al. [40] is particularly relevant. Their findings not only support the use of lower cut-off scores than the standard < 26 but also demonstrate that adjustments considering age, sex, and education are superior to simpler education-only corrections. Similarly, for the MMSE, the large-scale normative data from Mougias et al. [41] underscore the profound impact of demographic factors, showing that age and education together accounted for nearly 25% of the variance in MMSE scores in a healthy cohort. These studies provide strong external validation for our approach of using education-adjusted thresholds and reinforce our conclusion that such adjustments are essential for the accurate interpretation of cognitive screening tools.

The current results are consistent with growing evidence that supports the superior diagnostic performance of the MoCA over the MMSE in detecting early cognitive changes, particularly MCI. As emphasized by Breton, Casey, and Arnaoutoglou [33], the MoCA is more adept at capturing cognitive heterogeneity among older adults, enhancing its sensitivity to early and subtle impairments. Recent meta-analytical data reinforce this assertion. For instance, Malek-Ahmadi and Nikkhahmanesh [42] highlighted the MoCA’s higher diagnostic accuracy in identifying amnestic MCI, a precursor of Alzheimer’s disease. Complementing these findings, the comprehensive review by Oleksy et al. [34] concluded that the MoCA consistently outperforms the MMSE across a variety of clinical scenarios, particularly in cases where executive dysfunction, visuospatial deficits, or attention-related disturbances are predominant. Notably, their synthesis of over 60 studies showed the MoCA’s AUC ranging from 0.84 to 0.94 for MCI detection, compared to lower and more variable MMSE estimates, confirming the MoCA’s clinical superiority.

Educational level is widely recognized as a critical confounder in cognitive testing, affecting both raw scores and diagnostic thresholds. This issue was carefully addressed in the present study by implementing education-adjusted cut-off scores for both the MMSE and MoCA. As observed here and in prior studies [14,30,34], older adults with fewer years of formal education exhibited significantly lower cognitive performance, underscoring the role of education as a proxy for cognitive reserve. Xu et al. [43], in a large-scale meta-analysis, confirmed a clear dose-response relationship between years of education and reduced dementia risk. More recently, Lövdén et al. [44] have expanded on this evidence, demonstrating that longer formal education enhances resilience against cognitive decline across multiple domains, including working memory, processing speed, and verbal fluency. The incorporation of education-adjusted cut-off scores thus minimizes misclassification and enhances diagnostic accuracy, particularly when using the MoCA, which retained stronger agreement with CDR classifications in this study.

Adjusting neuropsychological test scores based on educational attainment significantly reduces misclassification bias in cognitive assessments. In this study, the MoCA maintained a stronger agreement with the CDR scale than the MMSE when education-adjusted cut-off scores were applied, reinforcing its superior discriminative performance. These findings are consistent with recent evidence from Kistler-Fischbacher et al. [45], who demonstrated that both MMSE and MoCA scores vary meaningfully according to years of formal education. In their large, multicenter European study, older adults with lower educational levels (≤12 years) consistently showed lower MoCA median scores compared to those with higher education. This underscores the need to account for educational background when interpreting cognitive screening results. Educational adjustment helps prevent overdiagnosis of cognitive impairment, particularly among individuals with limited formal education, thereby enhancing diagnostic accuracy and equity in diverse populations.

It is also important to highlight that educational attainment can influence cognitive biases during assessments, potentially leading to diagnostic confusion. Stanovich and West [46] observed that although more educated individuals generally performed better on cognitive assessments, they remained susceptible to specific cognitive biases that could complicate diagnostic interpretation. Therefore, the educational adjustments applied in this study were crucial not only for reducing false positives but also for mitigating the potential effects of such biases, improving the reliability of cognitive impairment screening [30,31].

Beyond educational attainment, age and functional capacity emerged as important differentiators between groups. Older adults with cognitive impairment exhibited poorer performance in both basic and instrumental activities of daily living (ADL and IADL). These functional deficits reflect the broader clinical implications of cognitive decline, emphasizing the critical role of integrating functional assessments into cognitive screening processes to enhance diagnostic accuracy [47,48,49]. Functional evaluations provide essential insights into the practical impact of cognitive impairment, underscoring the need for a multidimensional assessment approach.

Looking forward, the field of cognitive assessment is rapidly evolving with the integration of digital and remote screening tools administered via tablets and smartphones [50]. These technologies offer significant potential to increase accessibility, facilitate longitudinal monitoring, and streamline clinical workflows [51]. However, the fundamental principles highlighted in our study remain critically relevant. The superior sensitivity of instruments like the MoCA in detecting subtle deficits and the necessity of appropriate demographic adjustments are essential considerations for the validation and implementation of these new digital platforms. Ensuring that these emerging tools are both diagnostically accurate and equitable across diverse populations will be paramount to their success in improving early detection of cognitive decline [52].

Despite this study’s strengths, certain limitations must be recognized. The cross-sectional design precludes causal inference, restricting the interpretation to associative relationships. Future research should prioritize longitudinal designs to track cognitive trajectories over time and should integrate objective biomarkers, such as neuroimaging and fluid-based markers, to refine diagnostic conclusions. Furthermore, our hospital-based sample, with its specific health exclusion criteria and the exclusion of participants with fewer than four years of education, is not representative of the broader Brazilian population, which limits the generalizability of our findings. This reflects the well-documented ‘WEIRD population problem’ in neuropsychological research, where demographically constrained samples can lead to conclusions that may not apply universally [53]. Additionally, the sample included a higher proportion of females, which, while reflecting the demographics of the recruitment clinic and general healthcare-seeking behaviors, may limit the generalizability of the findings. Future studies should aim for more balanced samples to allow for robust analyses of potential sex-based differences in cognitive test performance.

Methodologically, we acknowledge that other neurological conditions (such as a history of traumatic brain injury or epilepsy) and developmental conditions (such as learning disabilities) were not formally screened for, which could potentially influence test performance. Similarly, although adjustments were made for key confounding variables, other potentially influential factors, including the cumulative burden of comorbidities and polypharmacy, were not systematically assessed. We also recognize that the reference standard itself, the CDR, may be subject to cultural biases, and while the rater was an experienced clinician, no formal cultural competency training was conducted. Finally, treating education as a purely quantitative variable, while a common practice, overlooks important qualitative and cultural dimensions that could influence test performance [29].

Finally, the scope of our analysis was limited. This investigation did not classify cognitive impairment into specific subtypes, such as amnestic or non-amnestic [54], nor did it distinguish among major or mild neurocognitive disorders (e.g., Alzheimer’s disease, vascular cognitive impairment, or frontotemporal dementia). Future studies should incorporate a more detailed categorization of cognitive impairment to achieve greater diagnostic precision.

## 5. Conclusions

The MoCA demonstrated greater sensitivity, specificity, and overall diagnostic accuracy than the MMSE for screening cognitive impairment among older adults, particularly when adjusting for education. These findings emphasize the critical need for appropriate selection and educational adjustment of cognitive assessment tools to enhance diagnostic accuracy. Additionally, integrating functional assessments into cognitive evaluations provides comprehensive insight into the clinical implications of cognitive changes, facilitating early identification, diagnosis, and intervention in neurocognitive disorders.

## Figures and Tables

**Table 1:** Sociodemographic, clinical, and functional characteristics of community-dwelling older adults with and without cognitive impairment (n = 90).

Variables	Cognitive Impairment		*p*
	Yes (n = 51)	No (n = 39)	
Sex, *n* (%)			
Male	17 (33.3)	11 (28.2)	0.603
Female	34 (66.7)	28 (71.8)	
Body mass index, kg/m^2^	28.0 ± 4.8	28.4 ± 5.2	0.692
Years of schooling	7.5 ± 3.1	9.6 ± 3.1	0.001
Beck Depression Inventory	10.0 ± 4.0	10.8 ± 6.1	0.627
Monthly income in minimum wages (MW *), *n* (%)			
Less than 2 MW	41 (80.4)	23 (59.0)	0.026
3 or more MW	10 (19.6)	16 (41.0)	
Chronic disease, *n* (%)	41 (80.4)	30 (76.9)	0.689
Number of prescription drugs	2.7 ± 2.0	2.9 ± 1.9	0.591
Activities of daily living	4.3 ± 1.0	6.0 ± 0.0	<0.001
Instrumental activities of daily living	21.9 ± 3.4	25.8 ± 0.8	<0.001
Mini-Mental State Examination	22.3 ± 4.0	26.5 ± 2.4	<0.001
Montreal Cognitive Assessment	15.3 ± 3.6	22.0 ± 2.8	<0.001

* MW: minimum wage (based on R$ 1212.00, according to 2022 data collection).

**Table 2:** Diagnostic performance of the Mini-Mental State Examination and Montreal Cognitive Assessment in screening for cognitive impairment using standard and education-adjusted cut-off scores (n = 90).

Neuropsychological Tests	Cognitive Impairment		*p*	κ	PPV (IC95%)	NPV (IC95%)
	Yes (n = 51)	No (n = 39)				
Mini-Mental State Examination (MMSE)						
Standardized cut-off scores						
Cognitive impairment (n = 37)	32 (86.5)	5 (13.5)	<0.001	0.479	0.865 (0.733–0.937)	0.641 (0.551–0.723)
No cognitive impairment (n = 53)	19 (35.8)	34 (64.2)				
Cut-offs adjusted for the years of education						
Cognitive impairment (n = 63)	44 (69.3)	19 (30.2)	<0.001	−0.132	0.698 (0.622–0.745)	0.741 (0.574–0.858)
No cognitive impairment (n = 27)	7 (25.9)	20 (74.1)				
Montreal Cognitive Assessment (MoCA)						
Standardized cut-off scores						
Cognitive impairment (n = 85)	51 (60.0)	34 (40.0)	0.013	−0.053	0.600 (0.571–0.628)	1.000 (0.478–1.000)
No cognitive impairment (n = 5)	0 (-)	5 (100.0)				
Cut-offs adjusted for the years of education						
Cognitive impairment (n = 58)	49 (84.5)	9 (15.5)	<0.001	0.746	0.845 (0.754–0.906)	0.938 (0.792–0.983)
No cognitive impairment (n = 32)	2 (6.3)	30 (93.8)				

Note: A negative kappa (κ) value indicates that the observed agreement was worse than the agreement expected by chance. PPV: positive predictive value; NPV: negative predictive value.

## Data Availability

The original contributions presented in this study are included in the article. Further inquiries can be directed to the corresponding author.

## Abbreviations

The following abbreviations are used in this manuscript:

ADLs	activities of daily living
AUC	area under the curve
BDI	Beck Depression Inventory
CAAE	certificate of presentation for ethical appraisal
CDR	Clinical Dementia Rating
EBSERH	Brazilian Company of Hospital Services
HU-UNIVASF	University Hospital of the Federal University of the São Francisco Valley
IADLs	instrumental activities of daily living
IBGE	Brazilian Institute of Geography and Statistics
MCI	mild cognitive impairment
MMSE	mini-mental state examination
MoCA	Montreal cognitive assessment
MW	minimum wage
NPV	negative predictive values
PPV	positive predictive values
ROC	receiver operating characteristic
SD	standard deviation
SE	standard error
STROBE	strengthening the reporting of observational studies in epidemiology
USA	United States of America
